# PARENTING ALL OVER AGAIN : HISPANIC “ABUELITAS” RAISING GRANDCHILDREN IN THE CONTEXT OF SUBSTANCE ABUSE DISORDERS

**DOI:** 10.1093/geroni/igz038.3230

**Published:** 2019-11-08

**Authors:** Bertha E Flores, Lyda C Arevalo-Flechas, Martha Martinez

**Affiliations:** 1 UT Health San Antonio, San Antonio, Texas, United States; 2 South Texas Veterans Heath Care System - Geriatrics and Extended Care Service, San Antonio, United States; 3 University of Texas Health San Antonio School of Nursing, San Antonio, Texas, United States

## Abstract

Around 7 million grandparents in the U.S live with grandchildren under 18 and 39% have primary caregiving responsibilities. In Texas, more than 313,499 children under age 18 live with a grandparent who is responsible for them. Hispanic grandparents are disproportionately more likely to care for grandchildren compared to non-Hispanic whites. In San Antonio, Texas, 36.6 % of grandparents are the primary caregivers for their grandchildren. Parental substance abuse disorders have been identified as one of the causes prompting child removal from parental custody. To understand the experience of custodial Hispanic grandmothers raising their grandchildren, three focus groups were conducted in English and Spanish by three bilingual investigators. Twenty-three grandmothers, mean age 60, caring for an average of 3.2 children, 2 months to 17 years of age participated in the focus groups. The narratives were transcribed and analyzed in the source language. The following overarching themes were identified: Family is family, parenting all over again, this is a struggle but a blessing, what did I do wrong? , fear of losing the children to foster care, I do not trust anybody with my children, financial and legal challenges, role captivity, aging as a limitation, and hope for the future. The findings from the study contribute to the body of knowledge necessary to foster urgent policy changes aimed at supporting these grandparents who feel unjustly treated by human services agencies and the legal system. Programs to support custodial Hispanic grandmothers need to be linguistically congruent and culturally competent.

